# A Case Report on Senear-Usher Syndrome

**DOI:** 10.7759/cureus.49268

**Published:** 2023-11-22

**Authors:** Shivani D Jangid, Bhushan Madke, Adarshlata Singh, Drishti M Bhatt, Arshiya Khan

**Affiliations:** 1 Dermatology, Venereology, and Leprosy, Jawaharlal Nehru Medical College, Datta Meghe Institute of Higher Education and Research, Wardha, IND

**Keywords:** crusted, acantholysis, autoimmune, bullae, erythematosus, pemphigus

## Abstract

Pemphigus erythematosus is an uncommon autoimmune bullous skin disorder with clinical, histological, and serological characteristics that overlap with lupus erythematosus and pemphigus foliaceus. The autoantigens are desmoglein 3, desmoglein 1, and desmosomal adhesion proteins in keratinocytes. When these bonds are disrupted, it causes acantholysis of keratinocytes, leading to the fluid collection between layers. Hence, the patient will present clinically with small flaccid bullae with crusting and scaling, mainly on the seborrheic areas. We report the case of a 21-year-old female presenting to us with multiple hyperkeratotic plaques, mainly on the seborrheic areas, including the face, chest, and elbows. The patient was evaluated further, and based on clinical and laboratory investigations, the diagnosis of pemphigus erythematosus associated with anti-double-stranded deoxyribonucleic acid (anti-dSDNA) and anti-nuclear antibody (ANA) positivity was made. The patient was then managed using immunosuppressant therapy, and the entire course has been detailed in this case report.

## Introduction

Pemphigus has four different clinical subtypes: pemphigus foliaceus, pemphigus vegetans, pemphigus vulgaris, and pemphigus erythematosus [1]. Cutaneous-limited lupus erythematosus (LE), intermediate LE, and systemic LE (SLE) are the three subtypes of LE according to symptom-based diagnostic classification. Localized (involving the malar regions) and generalized (relating to the broad morbilliform eruption) are terms used to characterize acute cutaneous LE lesions [2].

A study on "An Unusual Type of Pemphigus" was published in 1926 by Senear and Usher. Pemphigus erythematosus is an uncommon autoimmune bullous skin disorder that overlaps with LE and pemphigus foliaceus. The pemphigus lesions are flaccid bullae that rapidly rupture and turn into regions of crusted, oozing dermatitis or inflammatory papules with a thick, greasy, or even keratotic scale and crust over the trunk, mainly in the seborrheic areas. It is characterized histologically by acantholysis. Usually, the lesions involute on their own and leave behind dark-coloured patches [3].

Exposure to sunlight may worsen the condition. Pemphigus erythematosus typically has positive direct immunofluorescence (DIF) along the dermal-epidermal junction (DEJ) (reminiscent of lupus). It may have positive circulating anti-nuclear antibodies (ANA) in 30-80% of patients, even though the histopathological and clinical findings are similar to those of pemphigus foliaceus. Pemphigus erythematosus cases have been associated with anti-double-stranded deoxyribonucleic acid (anti-dsDNA), anti-Smith, anti-Ro, anti-Sjögren's syndrome-related antigen A (anti-SSA), and anti-ribonucleoprotein (anti-RNP) antibodies. Very few cases of pemphigus erythematosus have been related to anti-dsDNA antibodies, a highly specific SLE marker [4]. Treatment may include steroids like prednisolone, starting from the dose of 1 mg/kg/day and then tapered eventually, azathioprine, and other immunosuppressants [5]. There are many newer agents available, including biologicals. Rituximab, a monoclonal, chimeric anti-CD20 antibody, has been proven to have excellent efficacy, which helps in avoiding the side effects of corticosteroids. Tumor necrosis factor alpha (TNF-α) inhibitors can be used in recalcitrant pemphigus cases [6].

## Case presentation

**Figure 1:**
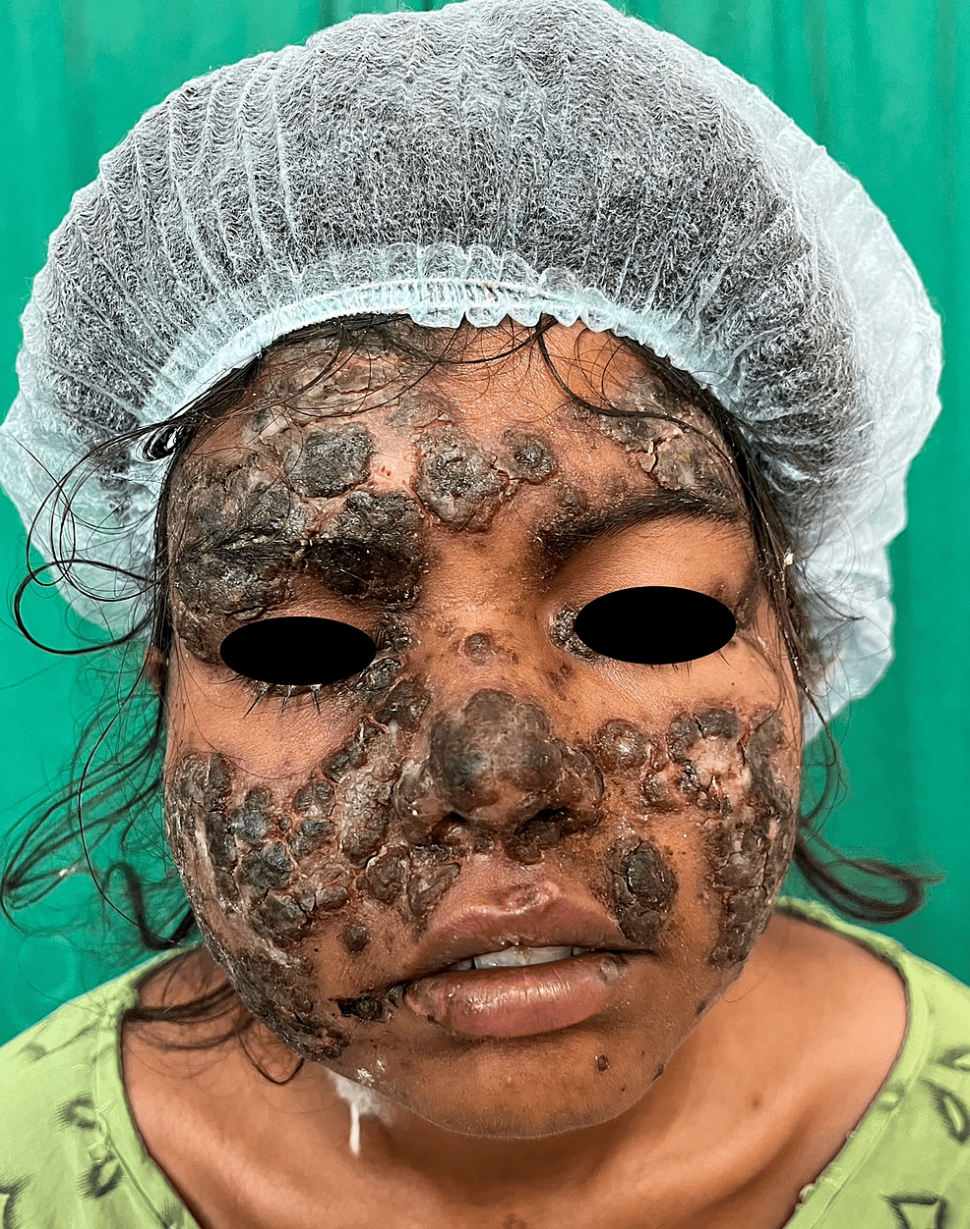
Multiple erosions present on seborrheic areas of the face topped with crusts

**Figure 2:**
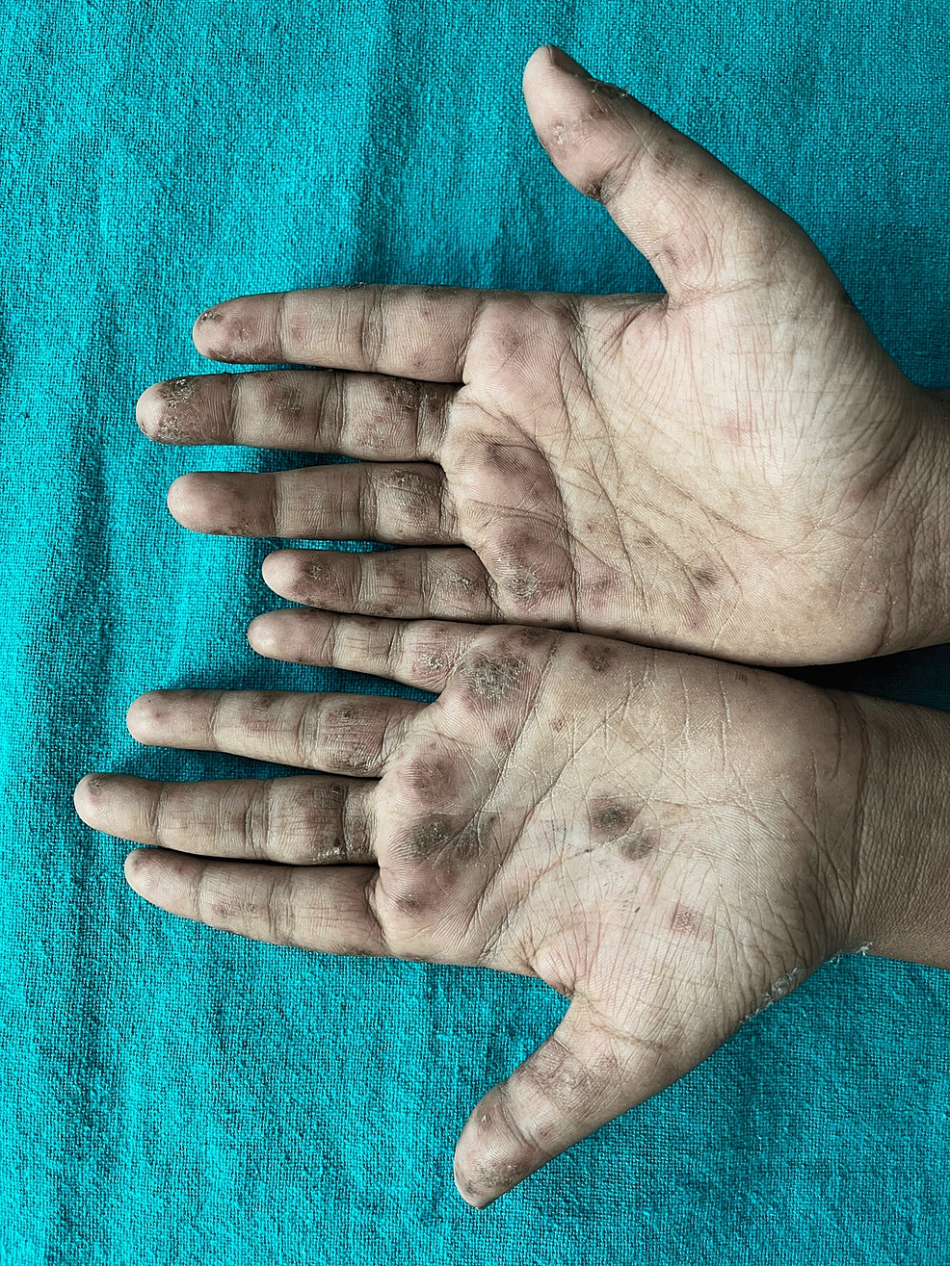
Multiple erythematous to violaceous macules and patches are present over bilateral palms. Few lesions are topped with scaling and crust

The case involves a 21-year-old woman residing in a rural area of Maharashtra, India. The patient came with chief complaints of multiple painful oral ulcers associated with difficulty in eating and chewing. One month later, she also developed many crusted plaques with an erythematous base initially over the malar region and ears, followed by similar lesions on the elbows and back, palms, and soles, which first appeared on the malar region on the face, followed by other sites on the body (Figure 1). There was occasional blood-stained discharge present from the lesions. There was evidence of crusting present on the eyelids as well. She had a low-grade fever and significant joint pain, limiting her daily activities. Cutaneous examination showed multiple hyperkeratotic plaques and crusts present over the erythematous base over the malar region, eyelids, and ears and multiple hyperpigmented plaques present over the bilateral elbows and back with the involvement of palms and soles (Figure 2). Her complete blood count was within normal limits except for low hemoglobin (8.5 g/dl). Her routine biochemistry testing for liver function and renal function test were within normal limits. Erythrocyte sedimentation rate (ESR) was raised (60 mm/hr), and ANA (6.311) and anti-dsDNA (1.28) by enzyme-linked immunosorbent assay (ELISA) were positive. DIF could not be done due to a lack of facilities.

**Figure 3:**
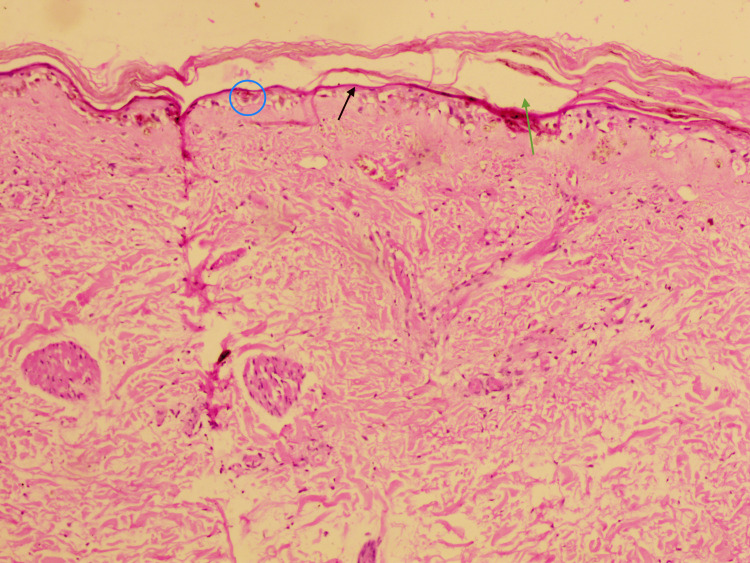
H&E staining under 10x magnification shows intraepidermal cleft (green arrow) with epidermal thinning with epidermal necrosis (black arrow) and vacuolar degeneration of the basal layer (blue circle) with congestion of the superficial dermal vasculature H&E: hematoxylin and eosin

A skin biopsy from one of the lesions on the right elbow showed epidermal thinning with epidermal necrosis and vacuolar degeneration of the basal layer. Superficial dermal vasculature shows congestion suggestive of acute apoptotic death of the epidermis (Figure 3).

**Figure 4:**
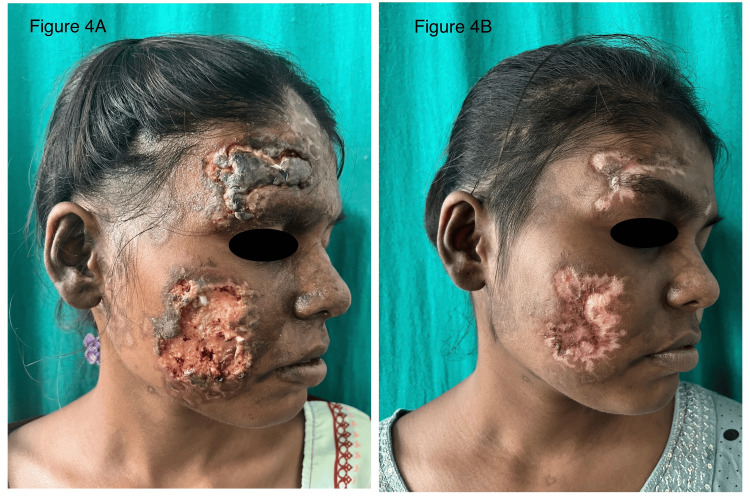
Lesions heal with ulcer formation at four weeks (Figure 4A) which eventually healed with scarring at eight weeks and hyperpigmentation (Figure 4B)

After admitting the patient, she was started on intravenous dexamethasone 8 mg once daily for seven days and later replaced by oral prednisolone at the dose of 1 mg/kg/day, which was then tapered to 0.5 mg/kg/day. Parenteral antibiotic amoxicillin and clavulanic acid 1.2 g twice daily was instituted for five days. Antimalarial drug hydroxychloroquine sulfate 300 mg was also prescribed. Dressing with normal saline-soaked gauze was done twice daily. For discharge in eyelids, dressing along with antibiotic eye drops was advised. After 15 days, the patient showed significant symptomatic improvement and was discharged on treatment and regularly followed up. Daily dressing and crust removal showed underlying ulcer formation, eventually healing with scarring and hyperpigmentation (Figure 4).

## Discussion

Amerian and Ahmed reported three male and one female pemphigus erythematosus cases. All four individuals had immunoglobulin and/or complement deposition at the DEJ on DIF examination of the perilesional skin. ANA testing was positive for all four patients. Different combinations of oral corticosteroids, topical corticosteroids, dapsone, and oral treatment were used to treat the patients. After four to 10 months of follow-up (mean, six months), three patients were in complete remission, and one was in incomplete remission. He concluded that for complete remission, patients with pemphigus erythematosus need considerably lower dosages of systemic corticosteroids [1].

Scheinfeld et al. discussed the case of a 44-year-old African-American female who presented with generalized skin eruptions. She was treated with oral prednisolone 80 mg once daily along with weekly intramuscular injections of gold 50 mg and showed dramatic improvement. Chief cutaneous findings of pemphigus erythematosus, along with positive deposits of IgG, IgM, and C3 in an intercellular pattern and along the DEJ, were suggestive of diagnosis. Histologically, suprabasal acantholysis may be present (as it is in the pemphigus foliaceus) [4].

Hobbs et al. proposed that pemphigus erythematosus is an autoimmune bullous disease having overlapping features of pemphigus foliaceus and LE. The classic presentation of pemphigus erythematosus is characterized by the overlapping features of SLE (DIF) and pemphigus foliaceus, including the deposition of multiple immunoreactions on a granular pattern (analogous to that seen in the lupus band) and the typical sub-corneal acantholysis present in pemphigus foliaceus [5].

Dick and Werth described various treatment options in patients with pemphigus. Although corticosteroids have significantly reduced mortality, morbidity is still present with corticosteroid treatment. Newer treatment options like biological and steroid-sparing therapies appear promising. Biologic agents like rituximab, a chimeric monoclonal anti-CD20 antibody targeting pre-B cells and mature B cells, prevent the formation of antibody-producing plasma cells. Infliximab, adalimumab, and etanercept (TNF-α inhibitors) have also been tried, but limited data is available. Patients receiving biologicals could taper their steroid dose and immunosuppressive agents [6].

Lyde et al. did an elaborate discussion on pemphigus erythematosus in a 5-year-old female who presented with blistering eruptions over her face, trunk, and extremities two days after a dental procedure associated with generalized erythema with scaling, erosions, and intact bullae present over the abdomen and extremities. After positive ANA and thorough investigations, she was treated with prednisolone 1 mg/kg/day and dapsone 50 mg/day and showed significant improvement [7].

According to Malik and Ahmed, eight patients with dual diagnoses showed no discernible SLE organ system involvement pattern. All had an immunologic disorder and a positive ANA; 75% had a haematological disease; 50% had renal involvement, photosensitivity, or a malar rash; and 38% exhibited signs of a neurological disorder [8]. Melchionda and Harman discussed an overview of cases of pemphigus, suggesting that tissue biopsy for histopathology and DIF remains the gold standard for diagnosis. A biopsy should be taken from a fresh, intact blister, including the edge. The role of indirect immunofluorescence (IIF) and ELISA is complementary [9].

Kasperkiewicz et al. pointed out a novel B-cell-depleting agent, veltuzumab. It is a monoclonal humanized anti-CD20 antibody that has shown promising results in patients refractory to other modalities. A single dose of subcutaneous veltuzumab resulted in complete clinical remission. Integrated treatment measures, including quality-of-life assessment in the clinical evaluation of patients, help in better treatment outcomes and modalities [10].

A descriptive table enlisting various cases of pemphigus erythematosus is given below (Table 1). 

**Table 1:** A descriptive tabular form of cases of pemphigus erythematosus ANA: antinuclear antibody; +: positive; dSDNA: double-stranded deoxyribonucleic acid; -: negative; Dsg1: desmoglein 1; ACA: anti-cardiolipin antibody; NR: nonreactive; IM: intramuscular

Case number	Author	Demography	Clinical manifestations	Serology	Histopathology	Treatment
1	Scheinfeld et al. [4]	44/female	Scaly, erythematous, and crusted plaques present on the face, trunk, and scalp	ANA+ dSDNA+	Dermal-epidermal junction: suprabasal acantholysis, marked hydropic degeneration	Prednisolone 80 mg + gold 50 mg IM weekly. Maintained on low-dose steroid and azathioprine 50 mg
2	Hobbs et al. [5]	37/female	Scaly, hyperkeratotic plaques over the scalp and face	ANA-	Epidermis: acantholysis; dermis: perivascular and interstitial infiltrate	-
3	Hobbs et al. [5]	44/female	Heavy scaling with underlying erythema	ANA- Dsg1+	Epidermis: acantholysis; dermis: inflammatory infiltrate	-
4	Hobbs et al. [5]	68/male	Raw erosive changes with some crusting over the trunk	NR	Epidermis: acantholysis; dermis: perivascular infiltrate	-
5	Hobbs et al. [5]	53/female	Scaly plaques with erythematous borders	ANA+ Dsg1+ ACA+	Epidermis: keratinocyte necrosis and alteration of basal cell layer; dermis: perivascular infiltrate	-
6	Hobbs et al. [5]	17/male	Vegetative plaques. Intact bullae and vesicles	NR	Epidermis: acantholysis	-
7	Lyde et al. [7]	5/female	Generalized erythema, scaling, and several bullae over the trunk and face	ANA+	Superficial acantholysis	Oral prednisone (1 mg/kg/day) and dapsone (50 mg/day)

## Conclusions

Pemphigus erythematosus is a clinical overlap of pemphigus foliaceus and LE. The diagnosis of pemphigus erythematosus can be challenging, and it involves a combination of various diagnostic methods. In addition to histopathological examination, various serological investigations, including routine blood tests and specific tests like ANA and anti-dSDNA, can aid in the diagnosis. However, a positive ANA is only detected in 30-80% of patients. It is imperative to come to a diagnosis in the case of pemphigus erythematosus, as pemphigus foliaceus affects only the skin. However, LE has a systemic involvement. Every dermatologist must become alert when they come across a patient with pemphigus foliaceus lesions on the malar area to diagnose pemphigus erythematosus since SLE, having systemic involvement, can be potentially life-threatening.
